# Comparison of the Effects of Different Forms of Nutrition Education on Adolescent Male Soccer Players

**DOI:** 10.3390/ijerph192113803

**Published:** 2022-10-24

**Authors:** Ziyu Gao, Sicheng Wang, Lianlian Peng, Lei Sun, Peng Qiu, Bingyi Bai, Qingqing Zhang, Junyu Wu, Yu Zha, Fenglin Zhu, Qirong Wang

**Affiliations:** 1 National Institute of Sports Medicine, National Testing & Research Center for Sports Nutrition, 1 Anding Road, Beijing 100029, China; 2 Sports Science College, Beijing Sport University, Beijing 100084, China; 3 Fuwai Hospital, Chinese Academy of Medical Sciences & Peking Union Medical College, National Center for Cardiovascular Diseases, Beijing 100037, China; 4 School of Kinesiology, Shanghai University of Sport, Shanghai 200438, China; 5 Beijing Football Association, Beijing 100050, China; 6 The First Hospital Affiliated to Wenzhou Medical University, Wenzhou 325000, China; 7 School of Sports Medicine and Health, Chengdu Sport University, Chengdu 610041, China

**Keywords:** youth soccer players, nutritional knowledge, nutrition education

## Abstract

The purpose of this study was to compare the educational effects on nutrition knowledge of two teaching methods targeting adolescent male soccer players through learning online from WeChat account articles (WeChat group) or taking classroom courses (classroom group). The study investigates whether such teaching methods can improve self-efficacy and nutrition knowledge for athletes. A total of 41 U15 (age 15) youth male soccer players, 21 in the classroom group and 20 in the WeChat group, participated in the experiment by receiving the same nutrition education separately for 12 weeks. An athlete nutrition KAP questionnaire and self-efficacy questionnaire were conducted before the intervention, immediately after the intervention, and 6 weeks and 12 weeks after the intervention. As a result, the nutritional knowledge score and the total score of the athlete nutrition KAP questionnaire in the classroom group increased significantly and were notably higher than those in the WeChat group. Self-efficacy scores improved significantly in both groups. In conclusion, the study showed that the level of nutritional knowledge of U15 male soccer players was mediocre, and both forms of nutrition education can significantly improve the level of nutritional knowledge and self-efficacy of the players. In comparison, the educational effect of classroom teaching is significantly greater and more consistent than that of learning from WeChat public articles.

## 1. Introduction

Positive nutritional status is of great significance to competition performance, training adaptability, fatigue recovery, and injury prevention for athletes. However, studies have found that many athletes lack nutritional knowledge and are unable to independently create a healthy and reasonable diet [1,2,3,4,5,6]. After researching the source of athletes’ nutritional knowledge, several studies have discovered that it mainly comes from coaches [2,7,8]. Family members, teammates, and the Internet are also important sources of information, as well as professionals and practitioners in the medical field [2,8]. Although coaches are an important source of nutritional knowledge for athletes, multiple studies have found that the nutritional knowledge level of coaches is mediocre [9,10,11]. The current research found that there were instances where athletes had fewer than three meals per day [12], and some ate breakfast irregularly [13]. Nutritional supplements are used by more and more athletes in order to meet the needs of body functions, improve exercise capacity, delay exercise fatigue and promote recovery [14,15,16]. However, some scholars have suggested that the prevalence of athletes’ use of nutritional supplements is not common [17]. There is room for improvement in the field of knowledge and application of nutrition for athletes.

Bandura [18] described self-efficacy as an individual’s perceptual ability in a specific field and the confidence to complete a certain task or behavior. Naturally, the formation of rational behavior cannot rely only on self-efficacy; one should also have the ability (knowledge, skills) to complete such behavior [19], and Bandura [20] believes that self-efficacy can promote the establishment of healthy behaviors. In their study, Larsen et al. [21] also proposed that as self-efficacy increases, results are expected to improve, and minimal or limited interventions may establish the improvement of self-efficacy as the main goal. Several studies have indicated that healthy nutritional patterns are associated with self-efficacy [22,23,24,25]. 

Studies have shown that imparting nutrition knowledge to athletes may have a positive impact on the formation of good eating habits and the improvement of sports performance [26]. Many athletes also agree that nutrition knowledge will promote changes in some of their own eating behaviors [3]. Devlin et al. [27] found that the intake of carbohydrates and protein of athletes with a high level of sports nutrition knowledge was closer to the recommended value. However, some scholars have proposed that it is necessary to continue to study and explore whether the level of nutrition knowledge of athletes can affect their dietary behavior [28]. Nevertheless, we can still see some positive trends, and we have not found that a high level of nutrition knowledge will have any adverse effects on athletes. Matin [29] also pointed out that athletes hope to learn more nutrition information to help themselves and promote the improvement of sports performance. Therefore, we can try to improve the diet behavior of athletes by increasing their nutrition knowledge.

As a basic form of nutrition education for athletes, traditional teaching is adopted by most researchers to impart nutrition knowledge to athletes through courses, lectures, etc. Matin [29], when conducting nutrition education for college athletes, adopted the method of combining presentation reports to teach nutrition to athletes and saw a positive impact. Bill et al. [30] sent nutrition information to the subjects through SMS to inform the athletes, but the intervention effect was not ideal. The athletes did not intend to change their dietary behavior, but they expressed interest in nutrition information.

Simpson et al. [31] used smart phone applications to provide nutrition education and feedback to athletes in his research. After six weeks of intervention, the athletes’ nutrition knowledge level and attitude toward accepting the suggestions of sports nutritionists were improved.

Currently, there are various different forms of nutrition education targeting athletes. With the development of information technology, the Internet is being used more and more for such a purpose. As an information communication medium, mobile phones are used to obtain information through different networks and social media outlets, such as WeChat, Facebook, and Twitter, which are also becoming more popular among young people. The form of education through emerging social media may be more convenient and accessible to young athletes as compared to traditional learning methods. At present, the comparison of the effects of different forms of nutrition education on athletes and the sustainability of the educational effects have yet to be studied. It is not clear which nutrition education method is more effective for athletes and how often they need to be re-educated. There is a lack of comparative research on the application of traditional teaching methods and emerging online multimedia methods of nutrition education for athletes. Therefore, this study is aimed at making comparative observations of the educational effects of different kinds of nutrition education (online and in class) on youth soccer players.

## 2. Materials and Methods

### 2.1. Study Subjects

The subjects of this study comprised two teams of U15 youth male soccer players from two clubs of the same level in the same region, with similar food standards and training intensity for their age group. No significant difference was found in the subjects’ demographic characteristics, questionnaire data or other baseline data. The method of grouping by club was adopted, dividing them into two groups due to the limitations of management and other factors in order to avoid mutual interference between different groups. One group was randomly assigned to receive nutrition education in the form of traditional classroom teaching, which was the classroom group; the other group learned through articles from online media (WeChat official account), which was called the WeChat group.

G*Power software (Version 3.1.9) was used to calculate the sample size; the effect size was selected as medium 0.25, α was selected as 0.05, the test power was 0.95, and the total sample size was 39 people. The subjects in this study were divided into two groups; the final subjects consisted of 50 people, with 25 in each group, keeping in mind the percentage of possible attrition (20%).

Subjects did not include those who met the exclusion criteria (exclusion criteria: gastrointestinal diseases such as eating disorders; use of medications that affect dietary intake; food allergies affecting dietary structure; having taken systemic nutrition courses; injuries that affect normal training). After the person in charge clearly explained the potential risks and benefits of the study and indicated that the study had been approved by the ethics committee, the subjects voluntarily signed the informed consent.

During the experiment, a total of 9 people (4 in the classroom teaching group and 5 in the WeChat group) were unable to complete the entire study due to injury, illness, special dietary needs (such as weight control), etc. A total of 41 people who fully participated in the experiment were studied, and the attrition rate was 18%, which is less than 20%.

### 2.2. Questionnaire Studies

#### 2.2.1. Athlete Nutrition Knowledge, Attitude, Eating Behavior Questionnaire (Athlete Nutrition KAP Questionnaire)

With reference to a large number of domestic and foreign research and literature, combined with the dietary characteristics of the Chinese population and the educational content of this study, the questionnaire compiled by Zeng Dan et al. [32] was adopted and amended in order to meet the needs of this study. The opinions of professional coaches and sports nutrition experts were considered, and the reliability and validity of the questionnaire were tested.

The athlete nutrition KAP questionnaire contains the following contents: basic information, nutritional knowledge (general and sports-related nutritional knowledge), attitudes towards dietary nutrition, and dietary behavior habits. The nutritional knowledge part includes knowledge about three major energy substances, vitamins, minerals, water, dietary fiber, dietary choices and meal-time choices for athletes in special circumstances, sports nutrition products and sports rehydration. The dietary nutrition attitudes detail the importance of proper diets for sports performance, the rational use of nutritional supplements, and attitudes toward participating in nutrition education programs. The part on eating behavior includes questions on the caloric intake of meals, snacks involving various categories of food, the effects of dietary intake before and after training or competition, and the use of fluids and nutritional supplements.

Regarding the scoring of the athlete nutrition KAP questionnaire, the basic information part is used as information collection and is not included in scoring, and the remainder comprises single-choice questions, except for questions 31–33 and 52, which are multiple-choice questions that are not included in scoring. The total score of the questionnaire ranges from 0 to 136 points, of which the total score of the knowledge part ranges from 0 to 20 points, the total score of the attitude part ranges from 0 to 50 points, and the total score of the behavior part ranges from 0 to 66 points. See Table 1 for details. 

#### 2.2.2. Questionnaire of Self-Efficacy

The self-efficacy questionnaire used in this study mainly refers to the questionnaire designed by Abood et al. [33]. The reliability and validity of the questionnaire was tested for young soccer players.

The self-efficacy questionnaire consists of 10 questions and uses a Likert 5-point scale. It involves self-efficacy regarding energy, the three energy sources, vitamins, minerals, exercise fluids, and meal timing. The options for each question consist of “strongly agree”, “agree”, “generally agree”, “disagree”, and “strongly disagree”, corresponding to 5 points to 1 point, respectively. The total score is obtained by summing the scores of each question. The total score ranges from 10 to 50 points. The higher the score, the higher the level of self-efficacy that the subject demonstrates.

In this study, the two questionnaires were completed four times: before nutrition education, immediately after education, and 6 and 12 weeks after education. The questionnaire was distributed to the subjects by researchers, who explained the questionnaire in detail and answered questions about the questionnaire raised by the subjects. After achieving the requirements for filling in the questionnaire, the subjects completed the questionnaire independently. The researchers proceeded to collect the questionnaire in a timely manner after the tests were finished.

### 2.3. Nutrition Education

Nutrition education program: Nutrition education lasted for 12 weeks. The classroom teaching group received one nutrition education class (combined with presentations) every week, which was explained by registered dietitians; the WeChat public account group read a nutrition education class pushed through the WeChat public platform every week. Educational articles were a combination of comics and text, with the content written by a registered dietitian. The two forms of nutrition education differed only in form; the content was the same, and the weekly learning time was 30 min.

The content of nutrition education included: two different forms of nutrition education related to energy sources, energy balance, the three major energy substances, vitamins, minerals, dietary fiber, sports rehydration, dietary principles for athletes, dietary characteristics of soccer events, sports nutrition, food safety, healthy eating behaviors, training nutrition, healthy weight gain and loss, and more.

In order to ensure the quality of education, a quiz was required for the subjects to complete after each learning lesson. In addition, the athletes in the WeChat group were allowed to consult licensed nutritionists about topics on nutritional knowledge. The WeChat public platform allows viewing the reading account and reading status in real time, and researchers could remind athletes in time to ensure participation. The classroom teaching team signed in to ensure participation.

### 2.4. Statistical Methods

The software Excel was used to summarize the data obtained from each test, and the software SPSS 21.0 was used for data analysis. The demographic data of athletes, the scores of the athlete nutrition KAP questionnaire, and the scores of the self-efficacy questionnaire were used for descriptive statistics and expressed in the form of mean ± standard deviation (M ± SD). A paired *t*-test was used to analyze the test data of the two groups before and after the intervention and at different time points after the intervention. The independent sample *t*-test was used to compare the differences between groups, and the repeated measures analysis of variance was used to analyze the persistence of the educational effect. *p* < 0.05 was considered statistically significant.

## 3. Results

### 3.1. Characteristics of Participants

A total of 41 subjects participated in the experiment, with 21 in the classroom teaching group and 20 in the WeChat group. The demographic characteristics of the 41 subjects were analyzed, and no significant difference was found between the two groups (*p* > 0.05), indicating that the absent 9 subjects did not have an impact on this study. The specific data are shown in Table 2.

### 3.2. Pre-Intervention Questionnaire Test Results for Nutrition Education

#### 3.2.1. Athlete Nutrition KAP Questionnaire Score

There was no significant difference between the two groups of athletes in total scores and the scores of the sports nutrition KAP questionnaire before exercise intervention (*p* > 0.05). Among them, as regards to nutritional knowledge, the average scores of the teaching group and the public account group were 12.33 points and 12.25 points, accounting for 61.65% and 61.25% of the full score, respectively. The average score of nutrition attitude in the teaching group and the WeChat group were 46.62 and 45.85, respectively (Table 3), accounting for 93.24% and 91.70% of the full score. The average score of diet behavior was 56.19 points for the teaching group and 55.90 points for the WeChat group, accounting for 85.14% and 84.70% of the full scores. The mean total scores of the two groups were 115.14 and 114.00, accounting for 84.66% and 83.82% of the full score, respectively (Table 3).

#### 3.2.2. Scores of Self-Efficacy Questionnaires

A questionnaire was used to examine the dietary self-efficacy of athletes. The results showed that the average score of the teaching group was 40.57 points and that of the WeChat group was 38.05 points. There was no significant difference between the two groups (*p* > 0.05) (Table 4). The mean scores of the two groups accounted for 81.14% and 76.10% of the full score, respectively.

### 3.3. Intervention Effect and Effect Comparison of Two Forms of Nutrition Education, and Comparison of Effect Persistence 

#### 3.3.1. Changes in the Scores of the Athlete Nutrition KAP Questionnaire before the Nutrition Education Intervention, Immediately after the Intervention, and 6 Weeks and 12 Weeks after the Intervention

The changes in the scores of the KAP questionnaire for athletes’ nutrition are shown in Table 5. Regarding the intervention effect, the nutritional knowledge score of the classroom group increased 20% after 12 weeks (*p* < 0.05). There was no notable difference in the scores of dietary nutrition attitude and eating behavior (*p* > 0.05), whereas the total score increased remarkably (*p* < 0.05). No significant difference was detected in the scores of each part of the WeChat group (*p* > 0.05), while the score of nutrition knowledge increased by 7% (Table 5).

With respect to the comparison of the effects of the two forms of nutrition education intervention, the results showed that the nutrition knowledge score and total score of the classroom group were significantly higher than the WeChat group after intervention (*p* < 0.05), but there was no major difference in the scores of dietary nutrition attitude and dietary behavior between the two groups (*p* > 0.05).

**Figure 1:**
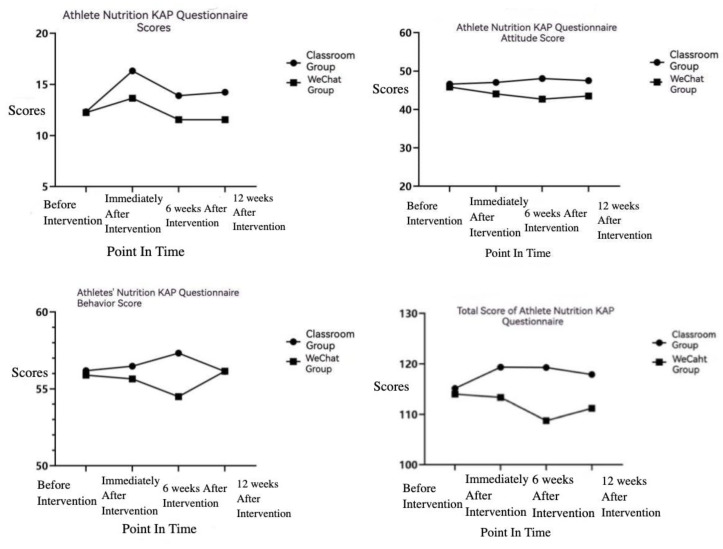
Athlete nutrition KAP questionnaire score trend.

Repeated-measures ANOVA was applied to analyze the long-term effects of the two nutrition education programs on the scores of the athletes’ nutrition KAP questionnaire. The results showed that the nutritional knowledge scores of the two groups fluctuated significantly over time (*p* < 0.05), but the interaction effect was minimal (*p* > 0.05). The attitude score of the two groups did not alter considerably over time (*p* > 0.05), while the interaction effect between time and groups was significant (*p* < 0.05). There was no significant fluctuation in the scores of the behavioral part of the two groups over time (*p* > 0.05), and the interaction effect between time and group was not significant (*p* > 0.05). The nutritional KAP questionnaire total scores of the two groups did not fluctuate significantly over time (*p* > 0.05); however, the interaction effect between time and group was significant (*p* < 0.05), and the changes between the two groups were significantly different. The change trend chart is shown in Figure 1.

#### 3.3.2. Changes in the Scores of the Self-Efficacy Questionnaire before the Nutrition Education Intervention, Immediately after Intervention, and 6 Weeks and 12 Weeks after Intervention

Regarding the intervention effect and effect comparison, the scores of the self-efficacy questionnaire were significantly improved in both the classroom group and the WeChat group after intervention (*p* < 0.05), and the mean value of the classroom group after intervention was moderately higher than that of the official account group, but there was no significant difference (*p* > 0.05) (Table 6).

**Figure 2:**
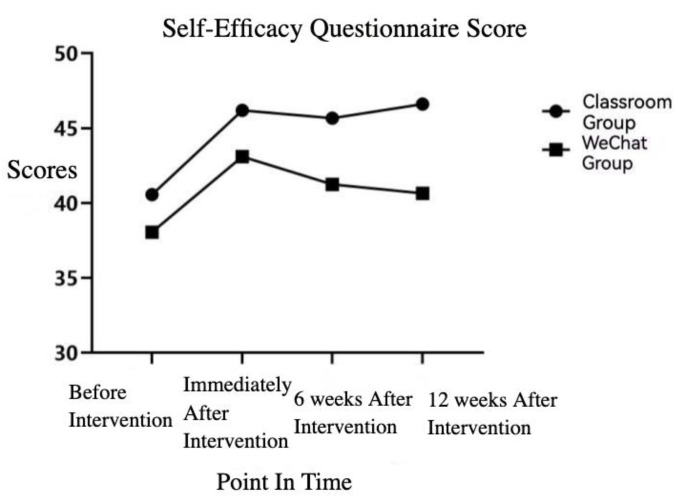
Self-efficacy questionnaire score trend.

The results of repeated measures ANOVA showed that the scores of the self-efficacy questionnaire fluctuated significantly over time (*p* < 0.05), but the interaction between time and group was inconsiderable, and there was no major difference between the two groups. The change trend chart is shown in Figure 2.

As regards the sustainability of the effects of nutrition education, the classroom group showed a marginal decrease 6 weeks after the intervention compared to immediately after the intervention, but this was nevertheless remarkably higher than the score before the intervention (*p* < 0.05). There was no significant difference at 6 weeks and immediately after the intervention, but both were significantly higher than that before the intervention (*p* < 0.05). The scores of the WeChat group 6 weeks after the intervention were slightly lower than immediately after the intervention, but both were significantly higher than before intervention (*p* < 0.05).

The results of the effect persistence comparison showed that there was no significant difference between the two groups 6 weeks after the intervention, while the score of the classroom group was significantly higher than that of the WeChat group 12 weeks after the intervention (*p* < 0.05).

## 4. Discussion

### 4.1. Nutrition Analysis of Young Soccer Players

A baseline survey was conducted in this study before intervention. The results of the athlete nutrition KAP questionnaire showed that the average scores of nutrition knowledge in the two groups were 61.65% and 61.25%, which indicates no significant difference between the group scores. The result is similar to that in the survey study of soccer players conducted by Devlin [27] and Abbey [8], with average scores of 56% and 55.2%, respectively. Lack of nutritional knowledge has also been found in several studies of adolescent athletes [28]. This was also confirmed in Zeng Dan’s [32] research on young soccer players in China. It can be surmised that the level of nutrition knowledge of athletes is required to be improved, and it is necessary to carry out nutrition education for athletes. 

The overall analysis found that athletes lack sports-related nutritional knowledge, which is roughly consistent with the low rate of 45.6% in the sports nutrition knowledge survey of Manore et al. [2]. This may be because most of the nutritional information that athletes encounter online is primarily generic knowledge about nutrition, which is rarely sports-related, resulting in a lack of knowledge of sports nutrition. Therefore, in addition to the explanation of general knowledge, attention should be paid to combining the characteristics of different sports when carrying out nutrition education for athletes.

A survey of sources of dietary advice for youth soccer players found that coaches, the Internet, friends or teammates were the most common sources of advice for athletes. Among them, a total of 29 people in the two groups (41 in total) had sought dietary advice from coaches, which is consistent with the findings by Devlin et al. [27] that coaches and websites are the main source of nutritional advice for athletes. In another study, it was found that the primary source of nutritional information for athletes was nutritionists, and a second opinion would be sought out from coaches and teammates [34]. In this study, the number of people seeking advice from a dietitian was low, possibly due to the fact that the club did not designate a nutritionist position, and athletes tend to choose the easiest way to obtain information. However, some studies have found that the level of nutritional knowledge of coaches is not commendable [9,10,11], which can cause young athletes to lose the ability to distinguish authentic information from that derived from the Internet, which contains an abundance of misinformation. Since it is not feasible to equip every youth soccer club with specialized nutritionists, it is paramount to carry out systematic nutrition education for young athletes and for coaches who may help promote nutritional proficiency in young athletes.

Regarding the survey on eating habits, the questionnaire scores of the two groups were 85.14% and 84.70%, which means these athletes have quality eating habits. In the study of Zeng Dan et al. [32], young soccer players scored 72.12% in this aspect. However, at the same time, problems emerged, as the frequency of consuming water was inadequate, with only 50% of the subjects drinking water regularly. The frequency of consuming water products in the provided dishes is slightly lower when athletes have meals in a cafeteria, which leads to the occurrence of this phenomenon. Therefore, providing nutritional advice to the cafeteria may be a wise choice to solve the issue. In addition, the survey found that the use of nutritional supplements by athletes is not frequent, which is inconsistent with the widespread use of nutritional supplements by athletes in the study of Wardenaar et al. [16] but is basically in congruence with the views of Muwonge et al. [17]. Moreover, Wardenaar et al. [16] studied elite and sub-elite youth competitive athletes, who were generally prevented from taking nutritional supplements because of the risk of doping. The management and distribution of nutritional supplements to athletes by clubs are inadequate at this stage, which may lead to decreased use of nutritional supplements.

The study found that young soccer players had higher scores on dietary nutrition attitude, with an average score of more than 45 points (90% of the full score) in both groups, indicating that young soccer players recognized the importance of dietary nutrition and were willing to correct their eating behaviors and attitude towards receiving nutrition education. This result is consistent with previous surveys of youth soccer players [32]. It also gives us more confidence in the importance and implementation of nutrition education for athletes.

The dietary self-efficacy of young soccer players in this study was sufficient, since the average scores of the two groups accounted for 81.14% and 76.10%, without a significant difference between them. This result is higher than Karpinski’s [35] self-efficacy survey score of athletes; the two groups accounted for 66.3% and 62.6% of the total score. However, further exploration is needed due to a lack of research on the dietary self-efficacy of different athletes in China.

### 4.2. Comparative Analysis of the Effect of Two Forms of Nutrition Education Intervention

After 12 weeks of nutrition education, the nutritional knowledge scores and total scores of the athletes in the classroom group increased significantly, but no considerable difference was detected in the scores of dietary nutrition attitudes and dietary behavior habits. The nutritional knowledge score marginally increased by 7%. The changes in the classroom group after intervention were consistent with the changes in the classroom teaching group after Zeng Dan et al. [32] conducted nutrition education for young soccer players. Although the level of nutrition knowledge was improved, their attitudes and behavior towards general diets and meals remained the same. The changes in nutrition knowledge and eating behavior scores in the WeChat group were about the same as in another study of similar athletes who received online article education, with only a 5.2% improvement in nutrition knowledge scores and a 0.7% change in behavior scores [34]. After the intervention, the nutritional knowledge score of the classroom group was significantly higher than that of the WeChat group, and there was no major difference in dietary nutrition attitudes and dietary behavior habits. The results of the self-efficacy questionnaire showed that the self-efficacy of the two groups of athletes increased significantly after the intervention, and the mean value of the classroom group was slightly higher than that of the WeChat group, without a significant difference. In Karpinski’s [34] research, both the online article-reading group and the athletes who received the interactive online nutrition education showed an improvement in self-efficacy after the 9-week intervention. However, there is no relevant research on the effect of nutrition education on athletes’ self-efficacy in China. 

Overall, the educational effect of nutrition education in the form of classroom teaching is better than that of learning nutrition through WeChat public account articles. Nutrition education in the form of reading online nutrition articles has certain effects, which are inconspicuous. However, nutrition education has no significant effect on the dietary nutrition attitudes and dietary behaviors of the athletes. This may be because the scores of these two aspects were relatively high before the intervention; the dishes provided by the cafeteria also affect eating habits.

### 4.3. Comparative Analysis of the Persistence of the Effects of Two Forms of Nutrition Education

The effect of nutrition education will gradually decline over time, despite the fact that nutrition education can improve the knowledge level of the athletes to a certain extent. The same mistake may resurface after a period of time, according to the study of Nowacka et al. [36], who conducted a 12-week follow-up survey on athletes receiving nutrition education. Combined with the data before and immediately after intervention, the overall change trend was analyzed. The results showed that the nutritional knowledge scores of the two groups fluctuated considerably over time. From the chart, we can recognize that after receiving nutrition education, the scores of both groups were improved before decreasing, and the change trend was similar. Further research on the persistent effect of nutrition education demonstrates that the influence level in the form of classroom teaching gradually weakened over time in terms of knowledge scores. After 12 weeks, it was significantly reduced compared to immediately after the intervention, but it was higher than before the intervention. No educational effect of online media learning was found 6 weeks after the intervention, and the nutrition knowledge level dropped to below pre-intervention levels. However, there were no significant fluctuations in the scores of dietary nutrition attitudes, dietary behavior habits and total scores. The change trends of attitude scores and total scores were different between the two groups; the classroom group had a trend of rising and then falling. Further exploration found that the score of the attitude part of the classroom group remained at a high level. Although the total score gradually decreased after the intervention, it was significantly higher than before the intervention, indicating that the educational effect of classroom teaching gradually weakened after education but still had effects in the 12th week. The WeChat group had no effect on these aspects, which was consistent with the previous comparison of the changes before and after intervention. 

There were significant changes in self-efficacy over time, and further research found that nutrition education in the form of classroom teaching had a certain educational effect in the 12th week after the intervention, whereas the online education remained effective after 6 weeks but deteriorated to pre-intervention levels after 12 weeks post-intervention. The continuous comparison of the two groups found that the knowledge part scores of the teaching group were significantly higher than the public account group in the 6th and 12th weeks after the intervention, as were the attitude part scores, and the total score of the athlete nutrition KAP questionnaire. The self-efficacy score was also significantly higher than that of the public account group, indicating that the educational effect of nutrition education in the form of classroom teaching is better than that of reading nutrition articles through WeChat public accounts. In contrast, when reading nutrition articles, key points are harder to comprehend and easier to neglect. Therefore, important details and topics should be highlighted and revised regularly when carrying out this form of education.

Overall, nutrition education in the form of classroom teaching and online reading for young soccer players improved the level of nutritional knowledge and self-efficacy of athletes but decreased over time, and the knowledge learned was gradually forgotten. Self-confidence may decline due to the loss of knowledge, so re-education is necessary. In addition, through the comparison of the two groups, it was found that the educational effect of classroom teaching is superior to that of reading nutrition articles online, which requires more frequent and earlier re-education. 

If the public account is used to push an article on nutrition education every week, it is suggested that re-education should be considered 6 weeks after the intervention to reduce loss of nutrition knowledge. It is suggested that nutrition education in the form of weekly classroom teaching should also be conducted again 12 weeks after the intervention to maintain education effects.

In addition to pushing articles, online media can also be used in athlete nutrition education in the form of online meetings, which may also be closer to traditional classroom teaching. When offline classroom teaching cannot be achieved, online meetings should be considered as a viable method.

In this study, nutrition education is conducted only through the teaching of nutrition knowledge, which does not guarantee the transformational effect of theoretical knowledge to actual dietary behavior, which is a limitation of this study. Nutrition education can combine the teaching of theoretical knowledge with the demonstration and correction of reasonable eating behavior and can further explore the educational effect through the dual intervention of knowledge and behavior. Future studies can further explore the educational effects of different types of nutrition education for athletes of different ages and sports.

## 5. Conclusions

The survey on the understanding of nutrition shows that U15 soccer players have a general level of nutritional knowledge. After completing two forms of nutrition education, the nutrition knowledge and self-efficacy level of U15 soccer players were significantly improved. Among the players, the educational effect of classroom teaching was significantly greater than that of online media (WeChat official account articles) in improving the level of nutritional knowledge. The effect of nutrition education in the form of classroom teaching is consistently superior to that in the form of online media material. Nutrition education in the form of online media should be considered for re-application in 6 weeks; nutrition education in the form of classroom teaching should also be re-applied in 12 weeks. 

## Figures and Tables

**Table 1:** Criteria for athlete nutrition KAP questionnaire.

Category	Questions	Score Criteria	Range of Scores
Nutritional Knowledge	1~20	correct = 1	0~20
		incorrect = 0	
Attitude towards Nutrition	21~30	A = 5	0~50
		B = 4	
		C = 3	
		D = 2	
		E = 1	
		No Answer = 0	
Eating Habits	34~43	A = 5	0~50
		B = 4	
		C = 3	
		D = 2	
		E = 1	
		No Answer = 0	
	44, 45	A = 1	0~10
		B = 2	
		C = 3	
		D = 4	
		E = 5	
		No Answer = 0	
	46, 47, 50	A or no answer = 0	0~3
		B = 1	
	48, 49, 51	A = 1	0~3
		B or no answer = 0	

**Table 2:** Approved study subject characteristics (N = 41).

Groups	No.	Age	Height (cm)	Weight (kg)	BMI (kg/m^2^)	Training Years	Education
Classroom	21	15.0 ± 0.4	172.7 ± 4.9	56.9 ± 6.0	19.1 ± 1.5	4.8 ± 1.6	Middle School
WeChat	20	15.0 ± 0.3	170.5 ± 4.7	55.0 ± 4.9	18.9 ± 1.1	4.6 ± 1.8	Middle School

**Table 3:** Athlete nutrition KAP questionnaire score (N = 41).

Group	No.	Nutrition Score	Attitude Score	Diet Habit Score	Total Score
Classroom Group	21	12.3 ± 3.8	46.6 ± 4.8	56.2 ± 4.3	115.14 ± 8.28
WeChat	20	12.3 ± 4.0	45.9 ± 4.7	55.9 ± 4.0	114.00 ± 9.02

**Table 4:** Self-efficacy questionnaire scores (N = 41).

Groups	No.	Scores
Classroom Group	21	40.57 ± 9.65
WeChat Group	20	38.05 ± 6.16

**Table 5:** Score changes of athlete nutrition KAP questionnaire (N = 41).

		Before Intervention	After Intervention	6 Weeks After	12 Weeks After
Knowledge Score	Classroom	12.33 ± 3.8	16.33 ± 2.9 *^※^	13.90 ± 3.7 *^#^^※^	14.24 ± 4.0 *^#^^※^
	WeChat	12.25 ± 4.0	13.65 ± 3.0	11.55 ± 3.5	11.55 ± 3.23 ^#^
Attitude Score	Classroom	46.62 ± 4.8	47.05 ± 5.4	48.05 ± 4.8 *	47.52 ± 4.8 ^※^
	WeChat	45.85 ± 4.7	44.05 ± 5.6	42.70 ± 12.3	43.50 ± 4.5
Behavior Score	Classroom	56.19 ± 4.3	56.48 ± 3.7	57.33 ± 2.7	56.14 ± 3.4
	WeChat	55.90 ± 4.0	55.65 ± 4.7	54.50 ± 11.3	56.15 ± 3.9
Total Score	Classroom	115.14 ± 8.3	119.86 ± 9.3 *^※^	119.29 ± 7.1 *	117.90 ± 9.7 *^※^
	WeChat	114.00 ± 9.0	113.35 ± 9.9	108.75 ± 25.5	111.20 ± 9.5

* Significant difference compared to before intervention (*p* < 0.05). ^#^ Significant difference compared to immediately after intervention (*p* < 0.05). ^※^ Significant difference compared with the official account group (*p* < 0.05).

**Table 6:** Changes in self-efficacy questionnaire scores (N = 41).

Groups	Before Intervention	After Intervention	6 Weeks After	12 Weeks After
Classroom	40.57 ± 9.65	46.19 ± 6.18 *	45.67 ± 5.97 *	46.62 ± 6.04 *^※^
WeChat Group	38.05 ± 6.16	43.10 ± 7.26 *	41.25 ± 8.27 *	40.65 ± 8.13

* Significant difference compared to before intervention (*p* < 0.05). ^※^ Significant difference compared to the official account group (*p* < 0.05).

## Data Availability

Not applicable.
